# The Brain–Cognitive Behavior Problem: A Retrospective

**DOI:** 10.1523/ENEURO.0069-20.2020

**Published:** 2020-08-07

**Authors:** György Buzsáki

**Affiliations:** 1 Neuroscience Institute and Department of Neurology, New York University, Langone Medical Center, New York, NY 10016; 2 Center for Neural Science, New York University, New York, NY 10003

**Keywords:** action, folk psychology, internalization, memory, perception, subjective terms

## Background

In 2001, I was invited to write a review for a prominent journal. I thought that the best way to exploit this opportunity was to write an essay about my problems with ill-defined scientific terms and question whether the dominant framework in neuroscience is on the right track. My main argument was that many terms in neuroscience are inherited from folk psychology and are often used in two ambiguous ways: both as the thing-to-be-explained (explanandum) and the thing-that-explains (explanans; e.g., “we have memory because we remember,” “we remember because we have memory”). These postulated terms are assumed to be entities with definable boundaries, and within this framework, the goal of neuroscience is to find homes and mechanisms for these terms in the brain with corresponding boundaries (I called this “the correlational approach”). I warned that a framework dictated by human-centric introspection might not be the right roadmap for neuroscience and argued that there should be another way of carving up the brain’s “natural kinds.”

A month later, I received the rejection letter: “Dear Gyuri, … I hope you understand that *for the sake of the journal* we cannot publish your manuscript” (emphasis added). One reviewer was very enthusiastic while the other strongly dismissive. I took a deep breath, put the issue on the back burner, and went back to the lab. Perhaps the harsh reviewer was right. Although I recognized the problem correctly, I failed to provide the right strategy to solve it. Yet, the issues I exposed in the manuscript kept bugging me, and I have since written two books (Buzsáki, 2006, 2019) as attempts to clarify my views and offer alternative strategies to explore brain-behavior relationships. In the intervening two decades, perhaps thinking along similar lines, other investigators have also addressed the explanandum-explanans issues (Krakauer et al., 2017; Brette, 2018; Cisek, 2019). By digging into the literature deeper, I also realized that many of “my ideas” had been considered already, often in great detail and depth, by numerous scientists and philosophers, although those ideas have not effectively penetrated mainstream neuroscience (Campbell, 1993; Hendriks-Jansen, 1996; Danziger, 1997; Lakoff and Johnson, 2003; Vanderwolf, 2003; Noë, 2006; Heinrich, 2017; Gomez-Marin and Ghazanfar, 2019). After re-reading the text of the rejected manuscript 18 years later, I found that many of the ideas presented there have become “contemporary.” Hence, I did an experiment.

I resubmitted the original manuscript (nearly two decades later!), and the current editor kindly agreed to send it out for review. Remarkably, three of the four referees recommended publication with minor revisions, whereas the fourth recommended major revision. They correctly pointed out that pitting theory against methods cannot be the right solution. I wholeheartedly agree with the reviewers that science is not just the art of measuring the world and that cutting-edge technique is not all there is to neuroscience. We have to be careful that new methods will not simply reveal more and more about less and less. Observations need to be organized into coherent theories to allow further progress. Yet, when caveats are recognized in a dominant model, alternative solutions oftentimes emerge by exploring the mechanical details of the substrate which the model is supposed to explain. Novel methods provide new windows on the same problems and can facilitate alternative interpretations. I have devoted several pages to this issue in my recent book (*The Brain from Inside Out*; Buzsáki, 2019). I still believe that promoting large-scale recording back in 2001 was a progressive idea, a method that has caught fire only recently. Yet, I support the view that technology cannot be the savior of the discipline. This latter sentiment is the key element of *The Brain from Inside Out*. Nearly all other comments and suggested revisions were based on papers published after 2001. Despite caveats and shortcomings of the original manuscript, it is interesting that just by virtue of time, or perhaps because of a shift in scientific perspective, the piece which was rejected 18 years ago became publishable.

Obviously, I did not want to revise the old text because by such revision would negate its historical value. Below is the original text and reference list (2001). The point of my exercise is to illustrate how dominant frameworks shared and defended by community consensus can oppose or welcome outside-the-box views. One just has to calculate the right time for publishing a particular idea. Of course, I do not expect that all my colleagues will agree with my views. Yet, providing alternative views is what makes progress in science.

## The Original Paper

The title of the original paper was “The brain-Cognitive Behavior Problem: Rethinking the Correlational Approach” with the following summary:

Although the goal of neuroscience is to understand how the brain generates behavior, current practice in behavioral/cognitive neuroscience appears to work in the opposite direction. Subjectively defined terms are selected as independent variables and their dependent (brain) variables are searched for. These terms, inherited from philosophy rather than derived from objective investigation of the brain itself, are assumed to be “real” entities. In such a framework, the major problems have been clearly outlined by previous introspection and the sole role of neuroscience is to discover and explain their mechanisms. In today’s practice, the reliability of brain-behavior correlations is judged by “inter-laboratory consensus” rather than by an independent assessment of the experimental error.

I suggest that behavioral-cognitive neuroscience should establish its own vocabulary, based on brain mechanisms, to formulate testable hypotheses. It should start with the brain and define descriptors of behavior that are free from philosophical connotations and can be communicated across laboratories, languages and cultures. A potential approach, using ensemble recording of neurons, is suggested to provide a quantified description of behavior and the associated experimental error.

## Introduction

“*Everyone knows what attention is. It is taking possession of the mind…”* (James, 1890)

The scientific investigation of brain mechanisms can be grouped into two major strategies: correlational and perturbational approaches. Independent of the approach, the only systematic strategy science possesses for the objective query of the simplest or most complex problem is hypothesis testing. The hallmark of scientific investigation is the formulation of a null hypothesis. Without a well-defined null hypothesis, no amount of work can lead to the rejection of the “null” and to the acceptance of alternative solutions. Progress in science is nothing more (or less) than perpetual rejections of hypotheses (Conant, 1950; Popper, 1959; Kuhn, 1970). Thus, the key element in scientific progress is the formulation of a well-defined null hypothesis that can be rejected by diligent work. Consider the shape of the earth. The null hypothesis to be tested here is the assumption that the Earth is flat. Once direct evidence is obtained that allows the rejection of the null (i.e., the Earth can be spheroid, ovoid, cylindrical, etc. but not flat), we can move to the next level. Now contrast this problem to issues like volition, imagination, emotions, hypnotic state, consciousness, or the mind. What is the null hypothesis of the mind and what would it take to reject it convincingly? I suggest that conceptual progress in cognitive behavior research is hampered by two fundamental problems. The first is that most of our terms to describe cognitive behavior have been inherited from philosophy rather than emerging from objective investigation of the brain itself (Bullock, 1970; Barnes, 1984; Vanderwolf, 1998). Although the ultimate goal of neuroscience is to understand how the brain generates behavior (i.e., how neuronal activity causes it), current mainstream behavioral/cognitive neuroscience appears to work in the opposite direction. Typically, we choose a subjectively defined term (e.g., volition, imagination, emotion) as the independent variable and look for dependent variables in the brain. This strategy is based on the assumption that the independent variable represents a real, objectively existing entity. More rarely, we begin with a distinct brain pattern (e.g., γ frequency oscillation) and search for external correlates from our existing vocabulary. I will argue that most of our behavior-related terms emerged before and independent of neuroscience, and there is little guarantee that these terms correspond to circumscribed brain mechanisms. I suggest that neuroscience, as any new discipline, should establish its own vocabulary based on brain mechanisms. It should start with the brain (independent variable) and define descriptors of behavior (dependent variables) that are free from philosophical connotations and can be communicated across laboratories, languages, and cultures. My second fundamental problem with brain-behavior research is that the reliability of brain-behavior correlations cannot be assessed independently. An assumed relationship proposed by one investigator cannot be quantitatively defined because its reliability depends solely on the subjective definition of the behavior term in question. In the jargon of statistics, no “error” measurement is provided. Without the objective measurement of error, comparison of conclusions obtained from different laboratories remains elusive. This is why I feel uncomfortable with terms like James’ “attention.” If in its definition one has to refer to its genus proximum, the mind (James, 1890), it causes more confusion than clarification.

## Windows on the Brain

There are only a handful of correlational techniques at the neuroscientist’s disposal to observe brain activity without seriously interfering with it (i.e., non-invasive methods). For studying the human brain, the major tools include electroencephalography (EEG; Berger, 1929), magnetoencephalography (MEG; Cohen, 1968), functional magnetic resonance imaging (fMRI; Ogawa et al., 1992), and positron emission tomography (Kinomura et al., 1996). Whereas EEG and MEG do have the temporal resolution needed to follow brain activity at the time scale of behavioral changes, the spatial resolution of scalp or epidural recordings in the depth of the brain is limited (Gevins et al., 1999). The remaining (“imaging”) methods have better spatial resolution, but to date, they are orders of magnitude slower than the speed of behavior changes. Furthermore, even if advantages of all these methods were combined into a single technique, they would still lack sufficient spatial resolution to trace the activity of neuronal ensembles (cooperative groups of individual neurons), the dimension believed to be responsible for coding information (Churchland and Sejnowski, 1988). It is unlikely that current imaging methods will ever reach the needed temporal resolution since, e.g., fMRI methods measure slow catabolic, vascular, and extracellular changes rather than direct electrical activity (Raichle, 1998; Logothetis et al., 2001). This “resolution problem” justifies animal experiments in brain-behavior research. In animals (and in limited cases in humans with parallel therapeutic goals), the recording probes can be inserted precisely into a given target, thereby increasing spatial localization. Multiple-site single unit/field recordings not only have the required spatial and temporal resolution but at the same time both inputs and outputs of neuronal ensembles can be monitored (Chicurel, 2001). Released neurotransmitters, ions, and other molecules in the extracellular space can be directly measured by *in vivo* microdialysis. Optical methods can survey large areas (Grinvald et al., 1988) or can zoom in to view dendrites and even spines (Denk et al., 1996) at least on the surface of the intact, living brain. These brain-derived objective measures can be correlated with ongoing behavior.

## The Problem with the Terminology in Brain-Behavior Research

Neuroscience is a young science (Kandel and Squire, 2000). Perhaps in no other discipline is fundamental research so well justified as in the research of the brain because so little is known about the operations of the circuits. Despite this justification, most contemporary brain-cognitive behavior research has the appearance of applied science. The main reason for this, I submit, is that neuroscience inherited its vocabulary from philosophy and psychology. Most of Western man’s thinking about the brain-mind problem takes its roots from Aristotle, British empiricism (Barnes, 1984; Locke, 1993; Hume, 2000), and to some extent Cartesian dualism (Popper and Eccles, 1977; Sperry, 1981; Szentágothai, 1984). In this general framework, the world is presented to the brain by sensory input pathways, and in turn, the brain acts on the body by its output motor pathways. However, even a short course of brain anatomy makes one wonder whether such an input-decision-output model makes sense in terms of the known wiring of the brain. Whereas there is no doubt that information from our sensory receptors does reach the cortex by way of the thalamus, the empiricist approach provides no clue why cortico-thalamic fibers are nine times more numerous than the ascending thalamocortical connections (Steriade et al., 1990). Furthermore, the weight of this ascending path is a midget compared with the heavyweight corticocortical connections (Szentágothai, 1978; Douglas et al., 1995). Thus, if we are to deduce and understand brain function from gross anatomy only, a possible conclusion is that the brain is interested in itself rather than the world outside (Llinás, 2001).

Let me illustrate the problem of existing terminology by using an imaginary example that typifies the popular strategy of brain-behavior experiments in current mainstream journals. Let us search for the brain sites (dependent variables; brain correlates) that are critically involved in “greediness” (independent variable; behavior) using imaging or other methods. Suppose that we find task-related changes in the measured variables in three anatomically defined structures. If one of them is the prefrontal cortex, the experiment can be taken as evidence that the prefrontal cortex is indeed needed for complex behaviors, such as greediness, as implicated by previous perturbation and correlation research. If all three active sites in our imaginary experiment are, say, subcortical structures, it is all the more interesting because their participation was unexpected in greed behavior. But slam on the breaks! The mere demonstration of the “involvement” of the prefrontal cortex or any other structure should not be sufficient for a serious scientific publication. The only thing we really learned is that in this particular experimental setup, three brain sites show changes in some dependent variables when the subject follows a particular set of instructions. Cause-effect relationship remains questionable for several reasons. First, it is possible that during greed behavior the heart rate, blood pressure, respiration, temperature, isotonic muscle tension patterns, or other unobserved intervening variables changed, which, in turn, were fully or partially responsible for the detected changes. Even if all possible hidden intervening variables are controlled for in an ideal experiment, it is still uncertain whether it is the afferents or the intrinsic activity of the three structures that are responsible for the observed changes (Logothetis et al., 2001).

Obviously, the technical issues in our imaginary experiment can be successfully addressed with further research. However, even if all technical problems are overcome, the relationship between the term greediness and its brain correlates will remain unsolved, even if the term is operationally defined. Others may argue that the test measured avarice, cupidity, covetousness, selfishness, rapacity, acquisitiveness, covetousness, meanness, skimpiness, stinginess, laconism, or other similar states rather than greediness per se. The reason for this predicted disagreement is the lack of an a priori contract (i.e., a well-defined null hypothesis) regarding the exact semantic content of the term “greed.” It is too vaguely defined to have the same meaning in every language and may refer to subtly different behaviors in different cultures, giving rise to potential errors of interpretation. I suggest that most terms used in contemporary brain-behavior science suffer from the same problems. We take it for granted that words with philosophical-psychological connotations reflect real entities and the cognitive behaviors these terms (vaguely) describe correspond to definable brain mechanisms. This is the strategy I refer to as applied research in behavioral/cognitive neuroscience. This approach tacitly assumes that the key problems to be solved by neuroscience have already been outlined by philosophy and psychology and only solutions are needed to these preexisting issues. To me, it is a bit like taking it for granted that the Earth is flat and at the center of the Universe. Science is needed only to explain why this should be that way.

Mapping function is an essential activity of brain research (Penfield and Jasper, 1954; Phelps et al., 1981; Merzenich et al., 1983). Nevertheless, the critical issue is whether the terms we borrow from philosophy and psychology reflect independent and circumscribed functions. One wonders whether our current brain map model is substantially less ridiculous than Gall’s cartoons of brain functions. Gall (1809) has been criticized not only because he arbitrarily superimposed some alleged functions on the wrong cortical areas but also because of the terms used in his phrenological maps. To his defense, those terms were considered the most fashionable in his days. Although today the priorities are on different alleged functions, the terms used in Gall’s days nevertheless survived, and numerous additional terms were created. Here is a short list of terms from some recently published papers: imagination, illusion, hallucination, humor, combat, love, religious belief, moral judgment, spiritual transcendence, attention, neglect, volition, self-determination, emotional engagement, motivation, drive, instinct, disgust, fear, panic, and rage. Do all these terms correspond to independent functions? Are behaviors described by related terms (e.g., various forms of attention) generated by similar or overlapping brain functions? A potential problem with any brain phrenology is that there are only a limited number of distinct brain areas (i.e., a dozen or so cortical systems, including the 52 architecturally defined Brodman cortical areas) but virtually unlimited number of presumed functions. Philosophy and psychology have accumulated so many brain function terms that they simply do not fit into our brains.

One may argue that such a broad top-down approach (one can call it “brain function library”) is useful or even essential. Suppose that in a coordinated international program (with a scale equal to or larger than the human genome project), all currently and previously used behavior terms are mapped onto the human brain. Obviously, numerous related and conflicting terms will be mapped onto the same (or different) brain structures. In the next round of (possibly even larger) effort, the functional similarities of the terms with identical or overlapping brain sites can be examined, and their essential features can be extracted. The final result of this matching/selection program is a new vocabulary with a limited number of terms that are brain mechanism-relevant. Of course, the issue is whether such an applied research approach is practical and the most efficient way to gain insights into the operation of the brain, given the finite resources.

The paradigm illustrated above dominates current animal research as well. Again, we select a favorite term (a less spooky one though than in case with humans to make sure that the assumed function does exist in the brain of the animal in question) and examine a brain correlate (dependent variable). To date, the latter is typically a single neuron in a single brain structure. This is done despite the recognition that information is encoded in the interaction among neuronal ensembles (Konorski, 1948; Hebb, 1949). Note that not only the independent variable (i.e., the behavioral term) but also the structure is often arbitrarily chosen or based on previous perturbation (lesion, stimulation, clinical) studies. Neurons in anatomically connected regions or in regions with suspected physiological links are recorded one at a time. There are several inherent problems with the single unit approach. For example, it provides no direct information about the temporal relationship of neuronal discharges at different locations, precluding the inference of cause-and-effect relationships or input-output transfer functions. In addition to these technical problems, the mega-issues to be addressed in the discussion of brain-behavior correlation are (1) how many experiments are needed to reach a consensus regarding the neuronal structures involved in the generation of a particular behavior and (2) how to agree on the physiological function of a given structure. These issues are not trivial considering the large variability that emerge from the use of various species, age, gender, testing apparatuses, unit isolation criteria, statistical tests, etc. I doubt that even the most sophisticated meta-analysis of all data deposited in an “animal brain function library” will yield an answer that would satisfy us all. We cannot make use of the data collected by our colleagues if we do not have an a priory contract (i.e., a mutually agreed null hypothesis) regarding the exact meaning of the behavioral variables. So does neuroscience have to stand by until behavioral-cognitive scientists establish a coherent and quantitative notion of terms we are examining at the cellular-synaptic level (such as James’ famous shortlist: attention, conception, imagination, reasoning, instinct, emotion, and will), or should we look for other alternatives?

## A Lesson from Hippocampal Research

In contrast to the top-down, deductive approach, neuroscientists less frequently take a well-defined physiological phenomenon (e.g., long-term potentiation) and examine the behavioral conditions (e.g., memory) that might match (correlate with) it. Even in this bottom-up or inductive approach, we routinely choose the dependent variables from the existing psychological vocabulary. The results are often conflicting because of the differing connotations of the preferred terms (e.g., γ frequency oscillation reflects perceptual “binding,” consciousness, arousal, or even slow wave sleep; Gray and Singer, 1989; Traub et al., 1999; Steriade, 2000; Llinás and Ribary, 2001).

**Figure 1.**
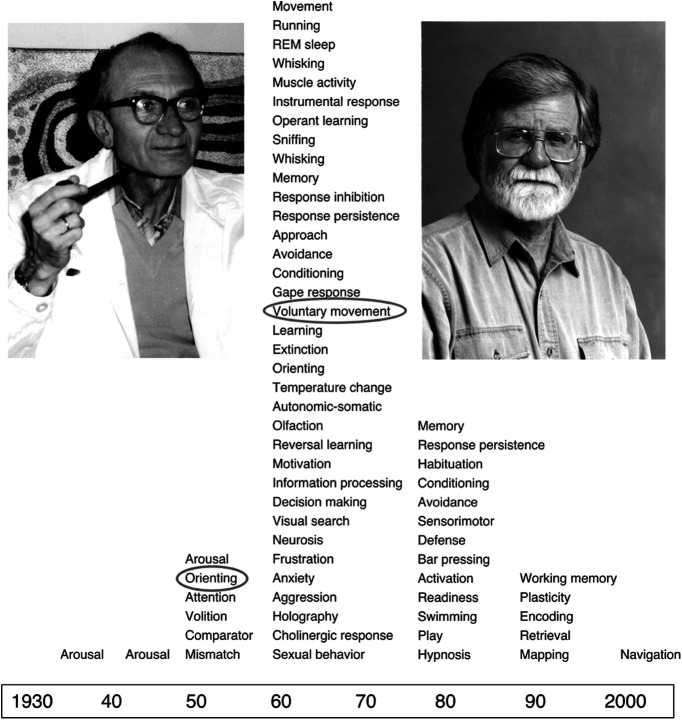
Time line of hypothesized behavioral correlates of hippocampal θ oscillations. Most correlates can be lumped as “sensory-attention” (input function) or motor output function. Endre Grastyán’s (left) “orienting response” hypothesis was the first, which was derived from observations in behaving animals (cat). The most influential hypothesis in the rat has remained the “voluntary movement” correlate by Cornelius (Case) H. Vanderwolf (right). Note the large variety of the hypotheses and their culmination in the1970s. The behavior (independent variable)–brain mechanism (dependent correlate) approach failed to produce a consensus on the behavioral significance of θ oscillations. Nota bene: This figure was subsequently published in *Hippocampus* [Buzsáki G (2005) Theta rhythm of navigation: link between path integration and landmark navigation, episodic and semantic memory. Hippocampus 15:827–840].

Let me illustrate the generality of these issues through a problem that I have closely observed throughout my neuroscience career. In my mentor’s laboratory (the late Endre Grastyán’s), we endlessly debated the behavioral-cognitive correlates of hippocampal θ oscillations (Fig. 1). In Grastyán’s view, hippocampal θ invariably correlated with the orienting response in the cat (Grastyán et al., 1959; Grastyán and Vereczkei, 1974), or in more general terms with input functions of the brain. Several other investigators agreed at least with the latter, broader view by suggesting that θ is a brain correlate of attention, focused attention, stimulus matching, perception, working memory, or similar terms (Adey, 1967; Vinogradova and Brazhnik, 1977; Bennett et al., 1978). However, Cornelius Vanderwolf (Fig. 1), studying the rat, challenged this broad view by stating flatly: hippocampal θ is a correlate of voluntary movement (Vanderwolf, 1969; see also Lopes da Silva and Arnolds, 1978). What else can be so diametrically opposite or remote in our psychological dictionary than perception and action (i.e., input and output)? So, as a student of Grastyán, I worked hard to reject what seemed initially as a ridiculous suggestion, i.e., that θ oscillations are brain correlates of voluntary class of movements (as classified by J. H. Jackson; see Taylor, 1958). However, every time we and others designed and executed experiments in an attempt to demonstrate the presence of θ episodes in the absence of overt movement, the critique bounced back to us: the animal was “planning” to move (Whishaw and Vanderwolf, 1973). At that point, all arguments became meaningless, since we could not explain neuronal mechanisms of planning or volition. By some trick of fête, I ended up becoming a postdoctoral fellow in Case Vanderwolf’s lab. By that time, there was hardly any term in the psychological dictionary that has not been claimed to be associated with hippocampal θ oscillations (Fig. 1). I had to face the reality that my best option to fame was to add yet another term to the already endless list of behavioral correlates of θ oscillations. I also realized that the final description of input processing (perception), attention, and short-term retention of the perceived world requires a volitional component, just like voluntary initiation of movement. In short, I concluded that the input meets the output at the site of the “will” by the “possession of the mind.” Of course, what really happened was the recognition that research entered the territory of “intentionality,” a label used to refer to the “substance” of all subjective mental activity by philosophers (Dennett, 1987). That other neuroscientists also recognized the magnitude of the problem, consciously or subconsciously, is illustrated by the quick disappearance of new theories about θ oscillations (Fig. 1). Thus, the problem of “θ-behavior” correlation that kept neuroscientists busy for half a century came to a gridlock. After 50 years and hundreds of experiments, there is no widely accepted term that would unequivocally describe behavioral correlate(s) of hippocampal θ oscillation.

There is a broader message in this endeavor, I believe. The skeptic-cynic generalization is that brain-behavior theories are built slowly (“incubation” time), proliferate, and then dissipate in a cyclic fashion. As Freeman (1995) notes, interest in the problem of consciousness has had a 50-year periodicity over the last few centuries. A more optimistic view is that such an exercise may lead to reformulation, refinement, and perhaps a lasting consensus of the competing behavior terms. Perhaps none of the terms that have been used to describe hippocampal θ captures the true content of the behavior. Nevertheless, most behavior terms advocated by researchers as the best correlate of hippocampal θ oscillation fall into the broad category of appetitive or preparatory behaviors. In contrast, operationally defined non-θ behaviors (such as immobility, grooming, eating, drinking, defecation, copulation, and slow wave sleep) correspond to the broad term of consummatory or terminal behaviors (Sherrington, 1906). In retrospect, this is after all not surprising. Oscillatory states may not represent any specific behavior but may permit that neurons of a given structure carry out computations necessary for generating specific outputs. Behavioral correlates of brain oscillations are therefore not expected to describe specific but rather broad classes of behaviors. But how do we get from broad behavior classes to specific ones and how can we relate the brain-derived specific behaviors to each other?

## How to Assess the Error in Brain-Behavior Correlation?

Because of the absence of quantitative methods, typically the validity of brain-behavior theories has been argued by a concordance approach. If the relationship between a chosen behavior and brain correlates appear similar in different laboratories, it is claimed that the theory is confirmed or validated, therefore it is “true.” For example, the most comprehensive and widely discussed description of hippocampal function is the “cognitive mapping” theory of O'Keefe and Nadel (1978). O'Keefe and Dostrovsky (1971) observed that single hippocampal pyramidal cells fire preferentially in a given part of the testing apparatus, and all places of the apparatus were associated with the discharge of a particular set of hippocampal neurons. Unlike neurons in the primary sensory or associational areas of the isocortex, hippocampal cells did not respond specifically to any particular feature of the apparatus or room cues. In fact, virtually any cue can be removed without affecting the firing pattern as long as some cues remained. The interpretation of these experimental observations was a major deviation from the empiricist thinking (Grastyán et al., 1959; Olds and Hirano, 1969; Ranck, 1973; Berger and Thompson, 1978). O'Keefe and Nadel resorted to another European philosophy and concluded that hippocampal activity represented “non-egocentric” or absolute (Kantian) space (Kant, 1788). During the past 30 years, numerous experiments, using similar testing conditions, supported the major claims of the mapping theory (Muller, 1996). During the same time period, only a handful of experiments claimed that hippocampal neurons respond primarily to stimuli other than the features of Kantian absolute space (Olton et al., 1979; Eichenbaum, 2000). Do these sporadic observations indicate that the Kantian space map theory of hippocampal function is not valid? According to the tradition of the scientific method, no amount of contradicting evidence can disprove an existing theory. Only a more comprehensive theory can replace an old one (Kuhn, 1970).

The sources of contradiction derive not only from behavioral factors (e.g., choice of controls) but also from the nature of the neuronal population tested. For example, it is generally believed that if majority of cells in a structure responds to variation in visual attributes then that area processes visual information (Hubel and Wiesel, 1977). However, a typical outcome of single unit experiments is this: 30% of neurons increase, 30% of neurons decrease their discharge frequency in response to a given experimental variable, whereas the remaining population does not respond specifically. Alternatively, a portion of neurons responds to one feature, another portion to a different feature, and the remaining population to a third feature (Wood et al., 1999). What is the conclusion regarding the function of the structure from which the neurons were recorded? The exact distribution of specific feature-related neurons often varies in different experiments and in different laboratories because of both technical factors as well as because of the lack of well-defined baseline and behavioral condition. These differences too often result in different theoretical conclusions. The crux of the problem in contemporary behavioral neuroscience, I believe, is the lack of agreed strategies and quantitative methods to provide error estimates of brain-behavior correlates. Without such error estimation, no amount of argument can decide which conclusion is right and with what probability. Estimation of the error should be derived from measurements that are not related to the observed behavioral variable.

Thus, is there any hope for a quantitative approach to brain-behavior function? I suggest that large-scale recording of neuronal ensembles offers an opportunity to quantify the reliability of brain-behavior correlations. The suggested approach is based on the premise that the variability of the spatio-temporal patterns of neuronal activity reliably correlates with behavioral variability (deCharms and Zador, 2000), because it is the spatiotemporal variation of neuronal activity that causes behavioral variability. If the exact timing and probability of spike occurrence in a single neuron is caused by the activity of its partners, then recording from sufficiently large number of partner neurons will allow us to reliably predict the occurrence of the spike. Therefore, spike occurrence in single cells can be correlated not only with an arbitrarily chosen behavior (e.g., spatial position) but also with the population dynamics of the recorded neuronal ensemble. Thus, for each spike train we can establish two correlations, one with behavior and one with the neuronal population. The difference in spike prediction from behavior and the neuronal ensemble can be regarded as the statistical error. The computation can be extended to all neurons recorded; thus, in essence, we can measure the match between behavior and the spatiotemporal dynamics of the population. For example, if the sole function of the population is to compute the Cartesian coordinates of space (O'Keefe and Burgess, 1996), the error will be small. On the other hand, if other non-monitored factors (e.g., smell, past experience) also affect the activity of the neuronal ensemble, the error increase will be proportional to the dominant role played by the non-monitored factors in the behavior paradigm. Working out the technical details of this general approach is costly and requires cooperation of cognitive-behavioral scientists, nano-engineers, computer scientists, and mathematicians. It is important to emphasize, however, that monitoring neuronal ensembles at the temporal resolution of neural signals is the best available assay to study brain mechanisms of cognitive behavior.

The quantitative assessment of the error between brain activity and behavior, in turn, will pave the way for the creation of an objective vocabulary of behavioral neuroscience. The emerging behavior terms, characterized from the viewpoint of the brain, can be communicated across laboratories and examined objectively. Once behavioral correlates are derived with objective methods in animals, we may begin to use these terms in search for structural-functional correlates in the human brain. This seems like a long way to go. Perhaps there are other (better) alternatives. However, without a concerted effort to define our behavioral-cognitive variables a priori and without measuring the experimental error involved, the brain-cognitive behavior problem may remain the territory of philosophy and psychology.

## Synthesis

Reviewing Editor: Christophe Bernard, INSERM & Institut de Neurosciences des Systèmes Decisions are customarily a result of the Reviewing Editor and the peer reviewers coming together and discussing their recommendations until a consensus is reached. When revisions are invited, a fact-based synthesis statement explaining their decision and outlining what is needed to prepare a revision will be listed below. The following reviewer(s) agreed to reveal their identity: NONE.

NA
